# Comparative transcriptome profiling reveals the role of phytohormones and phenylpropanoid pathway in early-stage resistance against powdery mildew in watermelon (*Citrullus lanatus* L.)

**DOI:** 10.3389/fpls.2022.1016822

**Published:** 2022-10-20

**Authors:** Vivek Yadav, Zhongyuan Wang, Yanliang Guo, Xian Zhang

**Affiliations:** ^1^ State Key Laboratory of Crop Stress Biology in Arid Areas, College of Horticulture, Northwest A & F University, Yangling, China; ^2^ State Key Laboratory of Vegetable Germplasm Innovation, Tianjin, China

**Keywords:** lignin biosynthesis, powdery mildew, phytohormonal crosstalk, peroxidase, watermelon

## Abstract

Yield and fruit quality loss by powdery mildew (PM) fungus is a major concern in cucurbits, but early-stage resistance mechanisms remain elusive in the majority of cucurbits. Here, we explored the comparative transcriptomic dynamics profiling of resistant line ZXG1755 (R) and susceptible line ZXG1996 (S) 48 h post-inoculation in watermelon seedlings to check precise expression changes induced by *Podosphaera. xanthii* race ‘2F’. Phenotypic responses were confirmed by microscopy and endogenous levels of defense and signaling related phytochromes were detected higher in resistant lines. In total, 7642 differently expressed genes (DEGs) were detected, and 57.27% of genes were upregulated in four combinations. DEGs were predominantly abundant in the KEGG pathway linked with phenylpropanoid biosynthesis, plant hormone and transduction, and phenylalanine metabolism, whereas GO terms of defense response, response to fungus, and chitin response were predominant in resistant lines, evidencing significant defense mechanisms and differences in the basal gene expression levels between these contrasting lines. The expression of selected DEGs from major pathways (hormonal, lignin, peroxidase, sugar) were validated *via* qRT-PCR. Detailed analysis of DEGs evidenced that along with other DEGs, genes including *PR1* (*Cla97C02G034020*) and *PRX (Cla97C11G207220/30, Cla97C02G045100 and Cla97C02G049950)* should be studied for their potential role. In short, our study portrayed strong evidence indicating the important role of a complex network associated with lignin biosynthesis and phytohormone related downstream mechanisms that are responsible for incompatible interaction between PM and watermelon resistance line.

## 1 Introduction

Watermelon (*Citrullus lanatus* L.) is a popular and economically important member of the Cucurbitaceae family. It is an important horticultural crop grown worldwide for its economic and nutritional importance (Davis et al., 2006; Wang et al., 2022b). A number of horticultural crops are found to be infected by powdery mildew fungus, but a majority of cucurbits are arguably the group most severely affected (Davis et al., 2007). The most prevalent, obvious, pervasive, and readily identifiable illness of cucurbits is certainly powdery mildew (Hückelhoven and Panstruga, 2011). Similar to other powdery mildew illnesses, it manifests as a whitish, talcum-like, powdery fungal growth on the cotyledon, petioles, stems, and leaf surfaces, with a rare exception of the fruits (Huckelhoven, 2005). According to (Zhu et al., 2018; Polonio et al., 2019), powdery mildew (PM) is a fungal disease brought on by *Golovinomyces cichoracearum* or *Podosphaera xanthii* in cucurbits. *P. xanthii* is thought to be the primary causal agent of powdery mildew on cucurbits and one of the most significant factors restricting cucurbit output. Watermelon powdery mildew is a major concern in major growing countries, including China, Turkey, India, and Iran, as it is in many other nations across the world (Yadav et al., 2021a). Watermelon plants are frequently affected by powdery mildew at all the development stages throughout the growing season, which causes necrotic lesions in the leaves and lowers the net photosynthetic rate, resulting in impaired plant growth and decreased fruit quality and overall yield (Keinath and DuBose, 2012). Contrarily, despite significant investments in disease prevention strategies, many fundamental facets of *P. xanthii* and plant pathogen interaction and clear molecular resistance mechanisms are still elusive. Currently, the primary strategy used to control PM infection is chemical prevention (Yuste-Lisbona et al., 2011; Cao et al., 2022). The generation and use of PM-resistant watermelon varieties is among the environmentally friendly, efficient, and most reasonable methods for controlling this disease, especially when compared to the drawbacks of chemical control (Kousik et al., 2019; Cao et al., 2021).

Traditional breeding has resulted in a number of isogenic lines with varying degrees of resistance to various PM races by transferring certain pathogen-resistant genes from wild resources to allied lines in many crops (Xin et al., 2012). The molecular processes underpinning the host-pathogen interaction and defensive responses in important agricultural plants have been better understood using PM-resistant lines as natural materials (Cao et al., 2022). They have additionally been praised for being the most reasonably priced and environmentally secure cultivars for disease prevention. It’s vital to research the PM defense mechanisms in watermelon to enable marker-assisted selection (MAS) of elite and broad-spectrum disease resistant cultivars (Li et al., 2020; Cao et al., 2022). The plant-pathogen interaction is a complex mechanism, and the resistance against infection in plants can be achieved through the rapid activation of multi-layered defense reactions.

**Figure 1:**
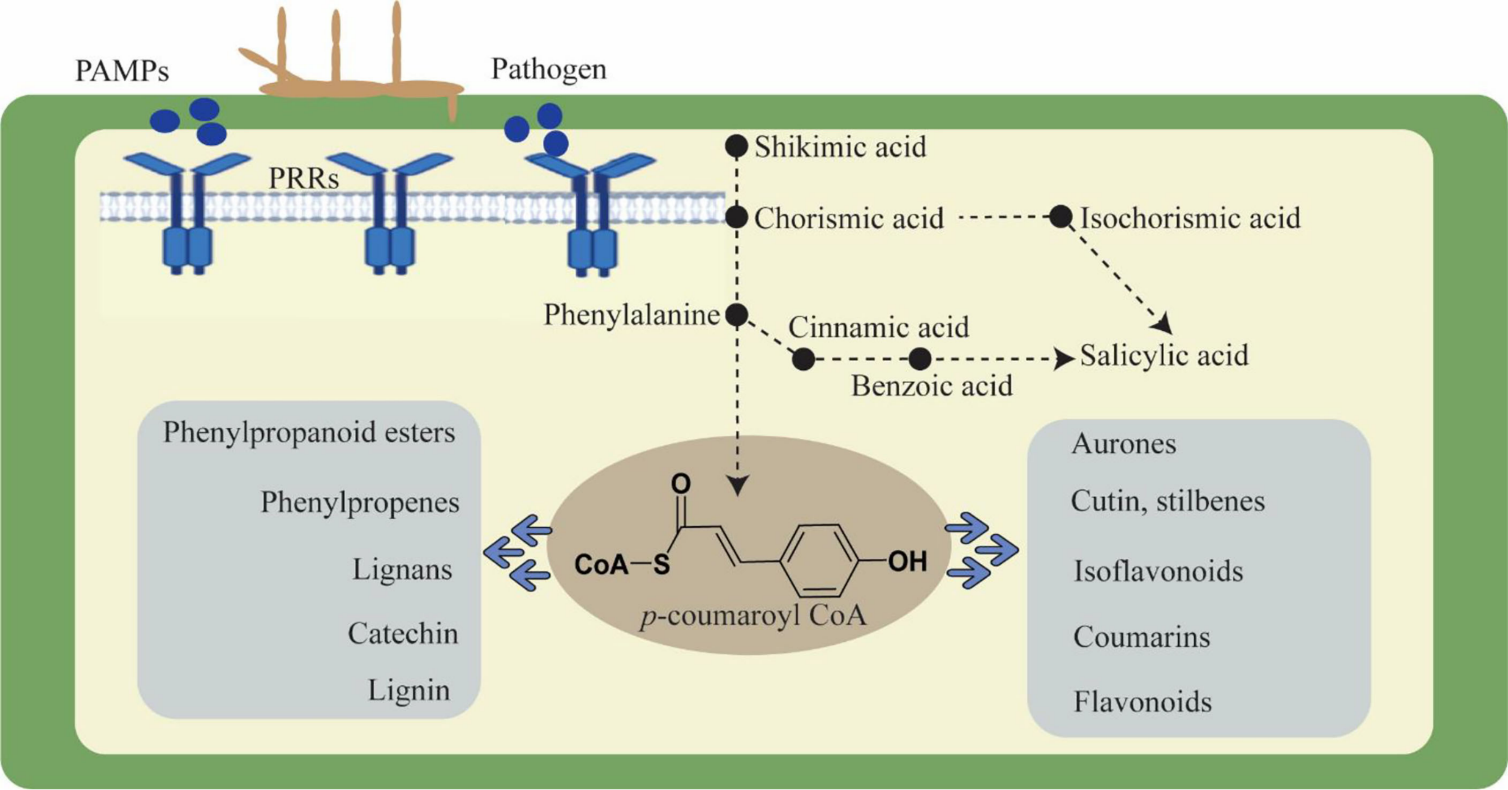
Cell wall dynamics during pathogen invasion and role of phenylpropanoid pathway in plant defense.

Moreover, plants react to these challenges in a dynamic and complicated manner. Modulating molecular processes, including the interaction of signaling chemicals like phytohormones, triggers a defense response (Collum and Culver, 2016; Wang et al., 2022a). These phytohormones regulate growth, development, and physiological activities in addition to defensive signals. Auxins, cytokinins, gibberellins, ethylene, jasmonic acid, brassinosteroid, salicylic acid, and abscisic acid are phytohormones that respond to stress by acting synergistically and antagonistically in a process known as signaling cross-talk (Benjamin et al., 2022; Fidler et al., 2022). These phytohormones cooperate harmoniously and react to environmental and developmental factors. All plant defensive responses are the consequence of the interaction of numerous genes and gene families that have been skilfully coordinated in a network. Through the regulation of genes, several phytohormones are recognized to have a significant part in practically every process. Furthermore, plants maintain homeostasis and adjust to environmental changes. Salicylic acid is an important phytohormone to induce defense responses against a number of biotrophic pathogens (Ngou et al., 2022; Yu et al., 2022). The SA mediated signaling pathway results in the biosynthesis of antimicrobial substances like phytoalexins and proteins related to pathogenesis and induces hypersensitive reactions (HR), which are frequently linked to programmed cell death in the infected area and limit pathogen colonization. In contrast, plants use the phytohormones ethylene (ET) and jasmonic acid (JA) to defend themselves against necrotrophs, and their reactions are comparable to those of being wounded (Kachroo and Kachroo, 2009; Kouzai et al., 2018). Phenylalanine serves as a key enzyme that can be converted to SA *via* conversion into cinnamic acid and benzoic acid, and SA is generated *via* the shikimic acid pathway (Shine et al., 2016). The PAL-catalyzed branch was formerly believed to be the only contributor to SA biosynthesis because of the stress-sensitive expression of phenylalanine ammonia lyase (PAL) and the decreased SA accumulation in response to PAL inhibitors (Bhuiyan et al., 2008). Phenylalanine, after conversion to cinnamic acid, turns into lead enzymes for basic flavonoids and lignin biosynthesis in plants. Hence, the role of the phenylpropanoid pathway becomes important in plant defense (Yadav et al., 2020). Additionally, lignification can also alter cell walls in a number of other ways, including making them more resistant to the enzymes that degrade cell walls, making them more resistant to the diffusion of toxins from pathogens to hosts, producing toxic precursors and free radicals, and entrapping pathogens (**Figure 1**). Lignin biosynthesis pathway genes are extensively found to be associated with local resistance against pathogens. For example, transient knock-down of certain gene encoding enzymes, such as caffeoyl-CoA O-methyltransferase (*CCoAOMT*), phenylalanine ammonia-lyase (*PAL*), cinnamyl alcohol dehydrogenase (*CAD*), and caffeic acid 3-O-methyltransferase (*COMT*) significantly reduced wheat basal immunity against the fungal pathogen *Blumeria graminis* (Bhuiyan et al., 2008). The penetration resistance in tobacco and Arabidopsis was also affected by down regulation or loss of *PAL* genes in plants against biotrophic and necrotic fungus. Similarly, recent reports evidenced that *TaCAD12* in wheat and *ZmCCoAOMT2* in maize confirmed quantitative resistance to multiple pathogens, indicating a vital role of lignin biosynthesis pathway genes in plant defense (Rong et al., 2016; Yang et al., 2017).

Core pathways and sensitive genes to biotic or abiotic stress have been widely identified using RNA sequencing (RNA-Seq), an innovative method for transcriptome research. It offers a precise measurement for transcript levels to disclose the response pathways to certain stimuli. For instance, by using comparative transcriptome analysis, disease stress-inducible pathways and their related genes have been studied in many important crops, including defense responses of mango against *Colletotrichum gloeosporioides* (Hong et al., 2016), tomato against *Phytophthora parasitica* (Naveed and Ali, 2018) and banana against *Fusarium oxysporum* (Kaushal et al., 2021). Transcriptomic research on some cucurbits such as melon and cucumber resilience to PM has recently gained popularity (Cao et al., 2022).

Profiling studies of gene expression have shown that considerable differences in gene expression are linked to the resistance response to a number of different plant pathogen groups. Comparative transcriptome analysis based on RNA-Seq is a crucial tool for discovering genes that are differently expressed in different plant part in response to environmental factors. This approach has been extensively used in studies on plant-pathogen interactions in some cucurbit crops like melon (Zhu et al., 2018) and cucumber (Li et al., 2016), but less frequently in watermelon, squashes, and other gourds. Recent studies have evidenced that comparative transcriptomic is an important tool for in-depth analysis. For instance, cucumber and melon were studied extensively among cucurbit crops for molecular processes underlying host resistance to the PM pathogen (Fukino et al., 2013; He et al., 2013; Xu et al., 2020). Compared to other cucurbits such as cucumber and melon, the details of research and knowledge are limited in watermelon. However, only a small number of quantitative trait loci (QTLs) and disease resistance genes linked to the response to PM in melon and cucumber have been discovered so far. Additionally, there is a paucity of knowledge regarding the genomic features and expression patterns associated with the watermelon response against PM race 2F. (Yadav et al., 2021a).

In this study, we have advanced previously available research and investigated transcriptional reprogramming triggered in resistant (ZXG1755) and susceptible (ZXG1996) lines with the aim of improving the understanding of the genetic control associated with host resistance by integrating analysis of various indicators in watermelon against the most prominent powdery mildew race 2F. For the first time, a comprehensive study of the monolignol genes and the crucial role of phytohormones for powdery mildew fungal defense against breaching the plant cell wall through transcriptome analysis combined with microscopy and phytohormonal profiling is presented. Our objectives were to assess the variations in the transcriptome between the two lines, identify the DEGs, and identify the major pathways implicated in the response. We also proposed a hypothetical mechanism behind the resistance mechanism against PM in the early stages of infection. This study gives a theoretical basis for making watermelon cultivars that are resistant to PM. It may also help us learn more about the molecular processes that make watermelon resistant to PM.

## 2 Materials and methods

### 2.1 Plant materials, race identification, inoculation and sample collection

Watermelon seeds of germplasm lines ZXG1996 and ZXG1755 were arranged from the Zhengzhou Fruit Research Institute, Chinese Academy of Agricultural Sciences, Zhengzhou. The seeds germinated on filter paper were seeded in 50 cell trays. Seedlings with cotyledons were planted into plastic pots filled with a mixture of peat and vermiculite (3:1) and growth was maintained in a growth chamber with 14/10 h light-dark at a temperature of 28/20°C (day/night), and a photosynthetic photo flux density (PPFD) of 600 μmol m^−2^s^−1^ (Yadav et al., 2021a).

A watermelon PM inoculation experiment was carried out using PM fungus *P. xanthii* race ‘2F’ routinely maintained on the sensitive watermelon lines under greenhouse conditions at the cucurbit germplasm innovation and genetic improvement center, Northwest A & F University, Yangling, Shaanxi, China (Yadav et al., 2021b). As per previous reports, melon differential lines were used for PM race confirmation. The conidial suspension (10^5^ spores/mL in 0.02% Tween 20) was prepared by young spores collected from infected leaves (Mandal et al., 2018; Mandal et al., 2020; Gao et al., 2021; Yadav et al., 2021b). Artificial inoculation was done by evenly spraying conidial suspension on the leaf surface of three-to-four leaf stage healthy seedlings. Seedlings used as mock inoculation controls were sprayed with sterile water containing 0.02% Tween-20. The leaf samples collected 48 hours after infection were immediately snap-frozen in liquid nitrogen and stored at -80°C until analysis. For ease of use, the samples were given the names S_CK/I to ZXG1996_Mock treated/PM inoculated, and the plants were called R_CK/I to ZXG1755_Mock treated/PM inoculated.

### 2.2 Microscopic observation and defense hormone quantification

Fully opened leaves harvested from mock and PM inoculated seedlings of watermelon were used for microscopic observation. Trypan blue powder dissolved in 40 ml of 1: 1: 1: 1 phenol/lactic acid/glycerol/water was used to stain the *P. xanthii* race ‘2F’ hyphae (Shine et al., 2016; Mandal et al., 2020). A sample for defense phytohormone measurements was extracted from a pool of four to five seedlings of both cultivars as described by (Liu et al., 2012) with some minor changes. Fine leaf powder (0.2 to 0.4 g of fresh weight) was extracted in 9 ml of 100% methanol and 0.2 ml of an internal standard solution (500 M o-anisic acid and 250 M p-hydroxybenzoic acid in 100% methanol). The methanol leaf powder suspensions were shaken for 1 minute (2,500 rpm) and centrifuged for 15 minutes at 6°C at 30,000 x g. The supernatant was vacuum-dried, resuspended in 2 mL of 5% (wt/vol) trichloroacetic acid, and extracted twice using 5 mL of ethyl acetate and cyclohexane (1:1, vol/vol). Prepared samples were used for quantification by HPLC-MS/MS.

### 2.3 Comparative transcriptome analysis

#### 2.3.1 RNA extraction, sequencing and transcriptome profiling

Three biological replicates of inoculated leaves for each treatment were used for RNA extraction and transcriptome sequencing. RNA isolation and high-throughput RNA sequencing (RNA-Seq) were conducted by Oebiotech Corp. (Shanghai, China). In short, total RNA extraction from frozen leaf samples was extracted using the mirVana miRNA isolation Kit (Ambion), and RNA integrity was evaluated on the Agilent 2100 bioanalyzer (Agilent Technologies, Santa Clara, CA, USA). The libraries were constructed using the TruSeq Stranded mRNA LTSample Prep Kit (Illumina, San Diego, CA, USA) according to the manufacturer’s instructions.

#### 2.3.2 Differentially expressed genes identification

In total, twelve high quality RNA samples from mock-treated and disease-inoculated leaves were prepared. On an Illumina sequencing platform (HiSeq 2500), 150-bp and 125-bp paired-end reads were produced from the libraries. After passing the quality inspection, sequencing was performed using an Illumina sequencer (Kim et al., 2015). Differential genes were screened according to the different multiple and different significance test results. Differential expression analyses among resistant and susceptible cultivars (S-I vs. S-CK and R-I vs. R-CK) were analyzed using the DESeq R package (1.10.1). The reads per kilobase of transcript per million mapped read (RPKM) normalization was used to examine the gene expression profiles. The counts of genes in each sample were standardized using DESeq, and the different significance of reads was tested using NB (Negative binomial distribution) (Love et al., 2014). The fold change presented in the differential expression data was used to indicate the ratio of expression between the treatments within a comparison. Genes with a fold change of > 1.5 and an FDR of < 0.05 were considered to be DEGs. The watermelon reference genome (97,103 V2) was downloaded from the website (http://cucurbitgenomics.org/organism/1) and paired-end clean reads were aligned to the reference genome using Hisat2 v2.0.4.

#### 2.3.3 Gene ontology and functional enrichment analysis

Gene Ontology (GO) enrichment analysis of identified DEGs were performed using Blast2GO v4.0. Kyoto Encyclopedia of Genes and Genomes (KEGG) (Kanehisa et al., 2007), enrichment analyses were performed using Gottools (https://github.com/tanghaibao/Goatools) and KOBOS v2.0 (http://kobas.cbi.pku.edu.cn/home), web bioinformatics tool to detect specific DEGs strongly associated with various metabolic and functional pathways.

### 2.4 Quantitative real-time PCR and expression validation

To confirm the authenticity of the transcriptome results, we selected a set of genes and performed qPCR. The primer sequences were designed using Primer Premier 5.0 software. The RNA samples used for the transcriptome were used for cDNA synthesis and utilized for qPCR-based expression analysis. In the data analysis for qPCR, the expression of candidate genes was normalized to that of the *Clactin* gene, a housekeeping gene in watermelon. The absence of non-specific products in the primer pair was verified using the melting curve. The assay for each selected gene was triplicated and quantification was evaluated using 2^-ΔΔ^Ct method. The primers used in the current study are listed in **Table S3**.

### 2.5 Data analysis

The data were analyzed, and differences between means were analyzed at different confidence level with IBM-SPSS v20 application. The graphs were made using ‘ggplot’ plot package in latest build of RStudio.

## 3 Results

### 3.1 Interaction phenotypes of *P. xanthii* race ‘2F’ on contrasting watermelon lines

**Figure 2:**
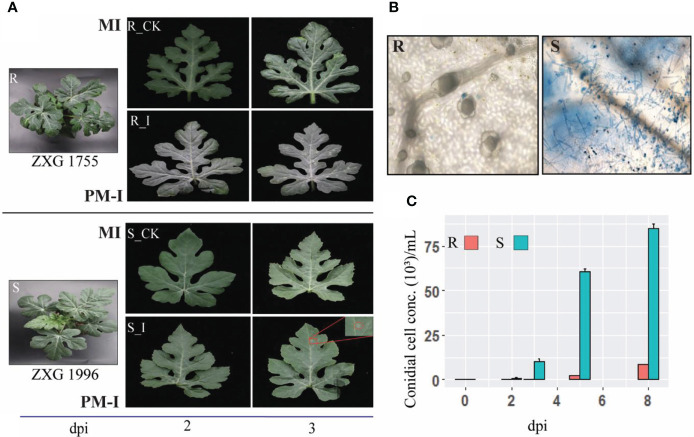
Interaction phenotypes of *P. xanthii* race ‘2F’ among mock and disease inoculated leaves of watermelon resistant and sensitive lines after 48(h). **(A)** Phenotypes of mock-inoculated (MI) and powdery mildew inoculated (PM-I) in resistant (R_CK/I) and susceptible (S_CK/I) cultivars of watermelon. **(B)** Post-invasion response and hyphal growth in different contrasting cultivars after post inoculations. *P. xanthii* race ‘2F’ hyphae were stained with trypan blue. **(C)** Bar graph depicting the number of PM conidials on the leaves of ZXG 1755/1996 lines on MI, PM-I, and at 5 and 9 dpi.

To examine the phenotypic response of resistant and susceptible lines, the extent of fungal growth was examined under the microscope. Analysis of phenotypes and microscopic observation revealed clear growth of PM hyphae on the leaf of susceptible lines (ZXG 1996). In contrast, no conidial growth and colonies were identified on the leaf surface of resistant lines (ZXG1755) (**Figure 2**). Moreover, the conidial growth was observed at different times, showing that the initial colonies in ZXG1996 (S_I) were visible after three days post-inoculation. However, there were no visual spores on the leaves of resistant plants (**Figure 2A**). Clear hyphal growth and spores of *P. xanthii* race ‘2F’ post-invasion were observed in ZXG 1996 (S_I) (**Figure 2B**). The conidial cell concentration examined in both the contrasting lines clearly showed significant growth of conidial cells on susceptible lines (**Figure 2C**). Conversely, the concentration of conidial cells was unaffected in resistant plants, and slight spores were measured after nine days post-inoculation in resistance lines. The results were in line with the previous reports of the high resistance and susceptible nature of watermelon lines used in the current study (Yadav et al., 2021b).

### 3.2 Quantification of endogenous phytohormones post-inoculation

**Figure 3:**
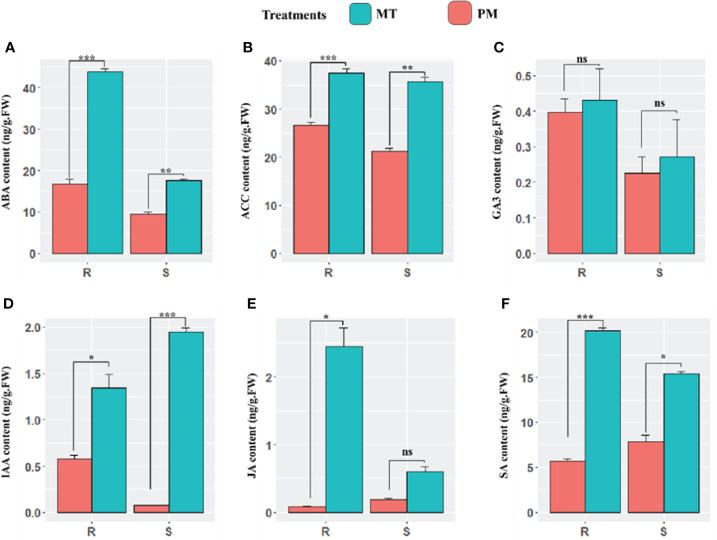
The endogenous content of major defense and signaling related phytohormones **(A–F)** including abscisic acid (ABA), auxin (AUX), ethylene (ACC), methyl jasmonate (MeJA), salicylic acid (SA), over the course of this study (mock-inoculated and PM inoculated after 48h phi) PM infection of susceptible and resistant watermelon lines, over early stages of invasion in leaves. The bars represent the standard error obtained from triplicate samples. *, **, and *** represent significant differences at P < 0.05, P < 0.01, and P < 0.001 (t-test), respectively. An unpaired t-test (P ≤ 0.05) was applied for the test of significance in hormone concentration in both contrasting lines. ns, not statistically significant.

In continuation, we comparatively examine the endogenous content of plant defense-responsive and signaling related phytohormones including abscisic acid (ABA), auxin (IAA), gibberellic acid (GA3), ethylene (ACC), salicylic acid (SA), and methyl jasmonate (MeJA) during the early stages of PM infection in both cultivars. At 48 h post-inoculation, the concentration of abscisic acid (ABA) in PM-infected plants compared to mock-treated plants significantly increased in the leaves of resistant and sensitive plants. However, the content in sensitive plants was less significant. 1-Aminocyclopropane-1-carboxylic acid (ACC), which is an immediate precursor of ethylene, showed higher content in resistance lines. The results revealed that there was no significant difference in the level of GA3 in both the lines. Moreover, a sharp increase in the level of auxin was observed in sensitive lines. The significant increase in auxin was reported in both lines. Overall, a higher concentration of SA was observed in ZXG 1996 in MI (S_CK) and PM-I (S_I) in comparison to ZXG 1755. Similarly, the endogenous level of JA was also examined, and the results showed a similar accumulation pattern to that of salicylic acid in the leaves of both lines (**Figure 3**).

### 3.3 RNA-seq, *de novo* assembly and annotation

During the compatible reaction on the leaves inoculated with the conidial solution, it has been reported that the post-invasion process undergoes five different stages of infection: conidia germination, haustorium formation, germ tube branching and hyphae elongation; conidia initiation and finally maturity of conidia. Previous studies in various crops, including different cucurbit early stages of infection (48 h) were considered to study early-stage compatible and incompatible interactions between watermelon and *P. xanthii* race ‘2F’. The detailed description of three biological repetitions is presented in **Tables S1** and **S2**. In this analysis, the referenced transcriptome sequencing of 12 samples was performed. A total of 85.56 G of CleanData was obtained, and a total of 47.44 to 51.41 million raw reads were obtained. The effective data volume of each sample ranged from 6.84 to 7.43 G, the Q30 base ranged from 90.06 to 92.56%, and the average GC content was 45.71% (**Table S1**). Moreover, multiple mapped reads ranged from 937547(1.96%)-to 1108197(2.22%) and total mapped reads (44911862(96.73%)-48043415 (98.14%)) were reported in all libraries, while reads varying from 94.67%–96.00% were uniquely mapped to the watermelon reference genome (97,103 V2). By aligning the reads to the reference genome, the genome alignment of each sample was obtained, and the alignment rate was 96.73-98.14% (**Table S2**). Based on the comparison results, protein-coding gene expression analysis was performed. Principal component analysis (PCA) of the transcriptomic data and correlation between samples are presented in **Figure 3A**. The differential screening was performed according to the expression levels of protein-coding genes in all samples used in the present study. There were four differential groups in total, and the number of detected differential genes was: 1037, 3169, 1869, and 1549, respectively (**Figure 3B**).

### 3.4 Comparative study of differential expression of assembled transcripts in response to PM post-inoculation

**Figure 4:**
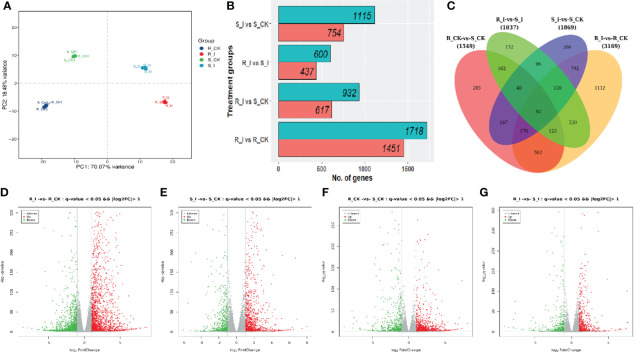
**(A)** Principal component analysis (PCA) of the replicated samples used for RNA-sequencing. All samples represented consistency in data among replicates. S/R_CK and S/R_I genotype factors showed variation among mock and diseases inoculated after 48 h. **(B)** Number of DEGs (p value < 0.005, statistically significant ≥ 2-fold), in different comparisons. **(C)** Venn graphs represent a cluster of DEGs from resistant and susceptible groups and interaction between the various treatment groups. **(D–G)** The volcano indicates the DEGs. Each dot in the figure signifies a particular gene. The green dot shows a significantly down-regulated gene, the red dot specifies a significantly up-regulated gene, and the dark grey dot is a group of non-significant differential genes.

To portray differentially expressed genes (DEGs) for the distinct phenotypic reaction of contrasting lines (**Figure 4**), gene expression levels were measured and DEGs were identified using R packages in order to study the differential expressions of genes responsive to PM infection. The analysis was carried out with all possible combinations, and four unique groups were created for comparative study. S_I *vs.* S_CK, R_I *vs.* R_CK, R_CK *vs.* S_I, and R_I *vs.* S_I are examples of sample comparative groups. The volcano distribution plots for up and down regulation comparisons among combinations are shown in **Figures 4D**-**G**. In total, 4774 DEGs were found in all comparison groups. Where the highest number of DEGs was 3169 (1451 up and 1718 down), followed by 1869 (754 up and 1115 down), 1549 (617 up and 932 down), and the lowest, 1037 (437 up and 600 down), DEGs were found in R_I vs. R_CK, S_I vs. S_CK, R_CK vs. S_CK, and R-I vs. S_I, respectively (**Figure 4B**). To get a more detailed analysis of DEGs, we created a Venn diagram. DEGs of mock-inoculated and PM-inoculated in both lines were distinguished (**Figure 4C**). After shorting, we found that there are a number of DEGs, some of which are unique, and some of which are commonly present between groups. The analysis evidenced that the maximum number of uniquely mapped DEGs, i.e., 1112, were found in R_I vs. R_CK, followed by 386, 285 and 152 in S_I vs. S_CK, R_CK vs. S_CK and R_I vs. S_I, respectively (**Figure 4C**). 792 DEGs were identified as commonly present in S_I vs. S_CK and R_I vs. R_CK, 162 DEGs were commonly mapped on R_I vs. S_I and R_I vs. S_CK, 96 common DEGs were discovered in the interactive region of S_I vs. S_CK and R_I vs. S_I, 162 DEGs were mapped on a common region of R_I vs. S_I and R-CK vs. S_CK and 92 common DEGs were identified in all possible combination groups (**Figure 4C**).

### 3.5 GO and KEGG analysis of identified DEGs in different groups

**Figure 5:**
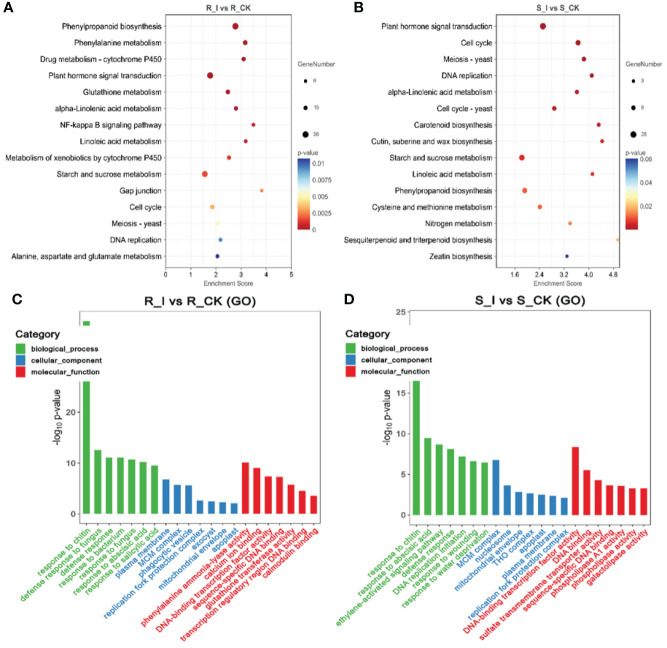
Pathway functional analysis of DEGs. The X and Y-axis represent enrichment factor and pathway names, respectively. A colored bubble represents p-value, and Rich Factor refers to the value of enrichment factor, which is the quotient of foreground value (the number of DEGs). **(A)** DEGs that were up-regulated in the R_I vs. R_CK groups. **(B)** DEGs that were up-regulated in the S_I vs. S_CK groups. **(C, D)** GO analysis of DEGs classified as biological, cellular, or molecular functions.

Analyses of the Gene Ontology (GO) and the Kyoto Encyclopedia of Genes and Genomes (KEGG) were carried out in order for us to determine which biological processes and functions are prevalent in DEGs. The identified DEGs were assigned to one of the three main GO categories: biological process, molecular function, or cellular component. The GO analysis revealed that in the biological process category, the majority of DEGs were associated with cellular processes, metabolic processes, and responses to stimuli in all combination groups. Under the cellular process category, the majority of genes were found to be associated with organelles, membranes, and extracellular regions in different comparison groups. Furthermore, activities associated with bindings, catalytic activities, and transporter activities were the major molecular function-related GO terms found to be associated with a majority of combination groups (**Figures 5C–D**). Notably, the study of the top GO terms showed that processes related to plant defense, including chitin response, defense response, and defense response to fungus-associated major biological processes were found in the R_I vs. R_CK group, which were different from S-I vs. S_CK. Interestingly, GO terms related to (phenylalanine ammonia-lyase activity) PAL activities were listed among major molecular functions in only the R_I vs. R_CK group (**Figure 5C**).

Furthermore, to study the biological pathways associated with PM inoculation in contrasting lines, DEGs were ammoniated by blast analysis against the KEGG database (Kanehisa et al., 2007). In order to get a depth analysis of the KEGG pathway enrichment analysis, we analyzed the detailed data and presented a table (**Table 1**) of DEGs using pathway annotations for various top events in each comparison group. In short, in response to PM invasion, plant hormone signal transduction (ko04075), phenylpropanoid biosynthesis (ko00940), glutathione metabolism (ko00480), phenylalanine metabolism (ko00360) and cell cycle (ko04110) are the major pathways associated with DEGs. A list of the top common pathways associated with DEGs in various groups is shown in **Table 1**. In short, overall phenylpropanoid biosynthesis pathways and phenylalanine-related DEGs were found in a majority of groups. When comparing the responses of the two cultivars, plant hormone and signal transduction were another pathway that shared a high number of DEGs, and the highest number of genes were reported in the R_I vs. R_CK and S_I vs. S_CK comparison groups (**Figure 5**). Other pathways, including phenylalanine metabolism, starch and sucrose metabolism, cell cycle, diterpenoid biosynthesis, linoleic acid metabolism, glutathione metabolism, and other pathways are also observed with a higher number of DEGs.

**Table 1:** List of top KEGG pathways associated with DEGs in various groups and number of up and downregulated genes in various comparison groups.

Pathway ID	Description	R_I vs. R_CK		S_I vs. S_CK		R_CK vs. S_CK		R_I vs. S_I	
		Up	Dn	Up	Dn	Up	Dn	Up	Dn
ko04075	Plant hormone signal transduction	28	23	24	13	13	9	8	7
ko00940	Phenylpropanoid biosynthesis	41	15	15	10	16	21	27	10
ko00360	Phenylalanine metabolism	19	3	4	2	4	8	11	5
ko00480	Glutathione metabolism	19	1	6	3	1	6	6	2
ko00500	Starch and sucrose metabolism	24	20	20	5	9	10	4	11
ko04110	Cell cycle	13	8	16	0	14	0	1	0
ko00592	alpha-Linolenic acid metabolism	11	5	8	1	2	4	4	0
ko00250	Alanine, aspartate and glutamate metabolism	9	3	2	3	0	4	1	2
ko04540	Gap junction	1	5	0	2	0	0	0	0
ko04113	Meiosis - yeast	10	4	10	1	6	1	1	1
ko03030	DNA replication	11	0	8	0	0	0	1	0
ko00040	Pentose and glucuronate interconversions	7	12	4	5	6	3	2	3
ko00220	Arginine biosynthesis	9	4	1	3	2	3	1	3
ko00903	Limonene and pinene degradation	2	1	0	0	0	1	0	0
ko00982	Drug metabolism - cytochrome P450	14	2	3	2	0	4	7	2
ko00400	Phenylalanine, tyrosine and tryptophan biosynthesis	7	0	1	1	0	3	1	2
	**% DEGs**	**67.976**	**32.024**	**70.5**	**29.48**	**48.667**	**51.333**	**60.976**	**39.024**
	**Total DEGs**	**225**	**106**	**122**	**51**	**73**	**77**	**75**	**48**

Upregulated (up) and down-regulated (dn).

### 3.6 Expression profile of DEGs associated to phytohormone signaling and transduction pathways

Plants have a wide variety of active and passive defense mechanisms at their disposal in the event that they come under attack by pathogens (Ausubel, 2005; Kumar et al., 2021; Benjamin et al., 2022). Active defense responses, which require *de novo* protein synthesis, are controlled by a complex and interconnected network of signaling pathways that primarily involve three molecules, ethylene (ET), jasmonic acid (JA) and salicylic acid (SA) and which results in the synthesis of pathogenesis-related (PR) proteins (Jones and Dangl, 2006; Sood et al., 2021; Fidler et al., 2022). In both compatible and incompatible PM reactions, KEGG pathway analysis found hormone-signaling-related genes that code for receptors and response factors. Comprehensive explanations are provided below.

#### 3.6.1 Differential expression pattern of auxin related genes during incompatible and compatible interaction

**Figure 6:**
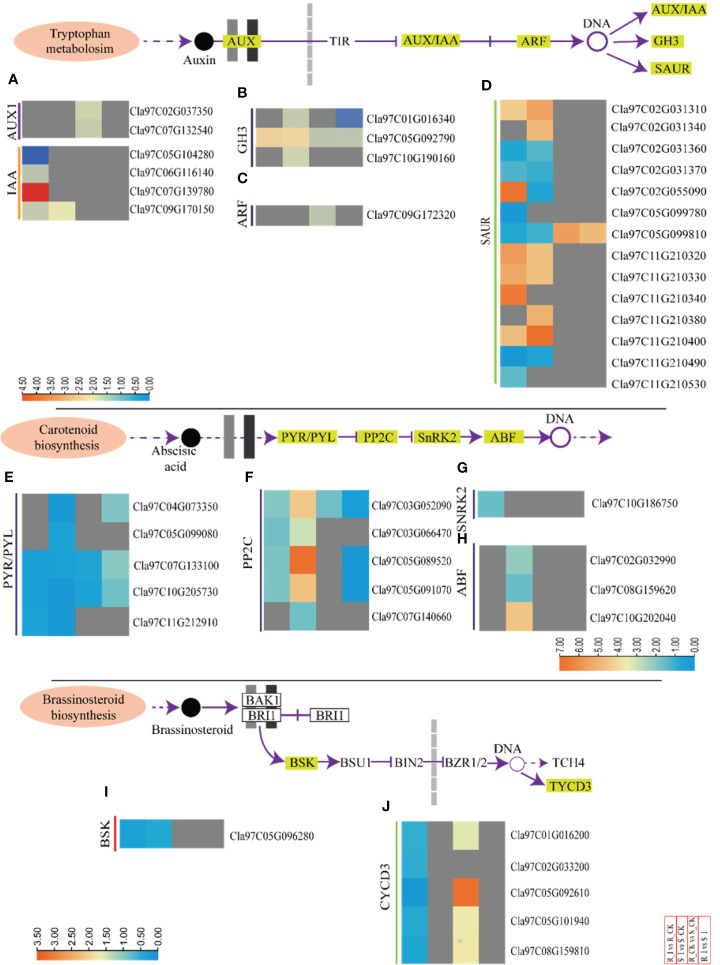
Transcriptomic analyses of incompatible and compatible interactions of the PM fungus in different watermelon lines. DEGs associated to auxin, abscisic acid and brassinosteriods from various combinations, such as groups R_I vs. R_CK, S_I vs. S_CK, R_CK vs. S_CK, and R_I vs. S_I, are presented in the form of a heatmap. The Log FC values corresponding to each gene in a group are used to generate a heatmap. The red and blue colours specify up-and down-regulated expressions. Genes related to IAA and AUX1 **(A)**, GH3 **(B)**, ARF **(C)**, and SAUR **(D)**, PYR/PYL **(E)**, PP2C **(F)**, SnRK2 **(G)**, ABF **(H)**, BSK and CYCD3 **(I, J)** are categorically shown in various combination groups. In the same way, heatmaps are used to show the genes of the abscisic acid **(E–H)** and brassinosteroid **(I, J)** biosynthesis pathways.

Here, we summarized the expression profiling of DEGs related to biosynthesis of auxin in all combinations (**Figures 6A–D** and **Table S3**). Representative DEGs include auxin influx facilitator auxin-responsive GH3 family protein (GH3); AUX1; indole-3-acetic acid inducible (IAA); auxin-responsive factor (ARF) and small auxin upregulated RNA (SAUR). SAUR-like auxin-responsive protein family related DEGs were filtered and analyzed.

In the R_CK vs. S_CK group, two *AUX1* genes (*Cla97C037350* and *Cla97C0132540*) were identified and both were upregulated. Moreover, one gene (*Cla97C07G139780*), out of four IAA identified genes, was up regulated after infection in resistant lines. One *GH3* (*Cla97C05G092790*) and one *ARF* (*Cla97C09G172320*) were upregulated in both groups. Fourteen SUAR genes were identified in DEGs. Out of them, six genes in R_I vs. R-CK and S_I vs. S_CK were upregulated, whereas one gene (*Cla97C05G099810*) was upregulated in R_CK/I vs. S_CK/I (**Figures 5A–D** and **Table S3**). In the compatible interaction group, the significant number of the auxin related genes were down regulated. For example, only one *IAA* gene (*Cla97C09G170150*), three *GH3* (*Cla97C01G16340*, *Cla97C05G092790*, and *Cla97C10G190160*), and one ARF (*Cla97C09G172320*) were downregulated (S_I *vs.* S_CK). Furthermore, the analysis of SAUR genes showed that six genes were upregulated, whereas five were down regulated. Notably, four SAUR genes (*Cla97C02G031310*, *Cla97C11G10320/30*, and *Cla97C11G210400*) were upregulated in both lines after infection in response to PM inoculation (**Figures 5A–D** and **Table S3**). The majority of *IAA* and *AUX1* genes in groups like R_I vs. S_I and R_CK *vs.* S_CK were not detected with significant transcript accumulation.

#### 3.6.2 Expression analysis of genes related to ABA (abscisic acid) and BR (brassinosteroid) biosynthesis

In total, 14 DEGs associated to the biosynthesis of ABA were observed in the majority of combinations (**Figures 6E–H** and **Table S3**). Among them, five DEGs of *PYR1*-like (*PYR*) were downregulated in all groups. The responses of PP2C genes were contrasting in resistant and sensitive lines. Four *PP2C* genes showed differential expression in both groups. Genes such as *Cla97C03G052090*, *Cla97C05G089520*, and *Cla97C05G091070* were upregulated in S_I vs. S_CK. Similarly, one ABF gene (*Cla97C10G202040*) was also ducted with a higher transcript after PM infection in sensitive lines (**Figures 6E–H**).

Protective activities of BRs against biotic stress have been documented based on field results (Bartwal et al., 2013; Yu et al., 2018). In the current study, seven DEGs associated with BRs pathways were identified. For example, all the identified DEGs were found upregulated in the R_CK vs. S_CK group, whereas only one detected plant brassinosteroid-signaling kinase (BSK) (*Cla97C05G096280*) was downregulated in resistant and sensitive groups after PM infection. No DEGs were detected in the R_I vs. S_I groups. In R_I vs. R_CK, the majority of *CYCD3* genes were down regulated (**Figures 6I, J**).

#### 3.6.3 DEGs related to phytohormones and defense signaling

**Figure 7:**
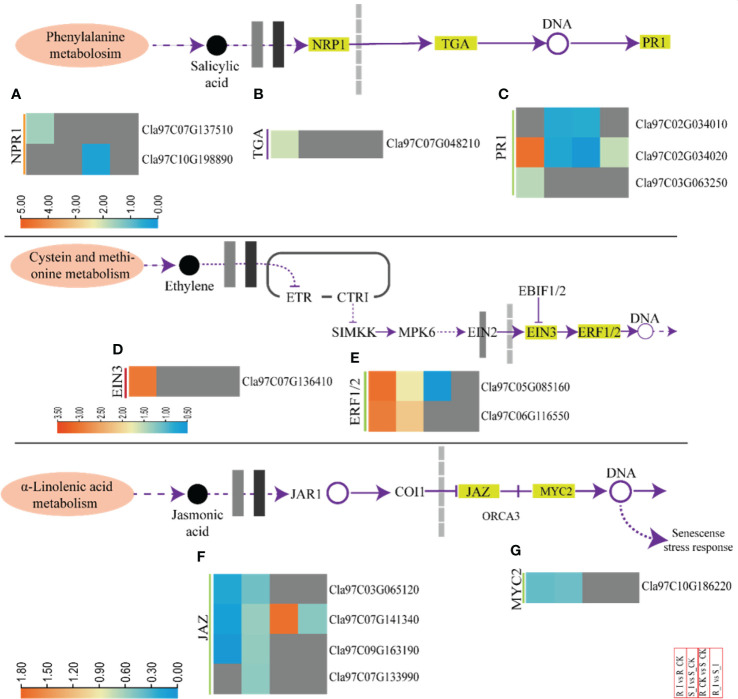
A heatmap of the genes related to phytohormone defense related activities with differential expression after PM infection in susceptible and resistant watermelon lines, over early stages of invasion in leaves. A heatmap shows DEGs from the NPR1 **(A)**, TGA **(B)**, PR1 **(C)**, EIN3 **(D)**, ERF1/2 **(E)**, JAZ **(F)**, and MYC2 **(G)** gene families from the different comparison groups R_I vs.. R_CK, S_I vs.. S_CK, R_CK vs.. S_I, and R_I vs.. S_I. The Log FC values corresponding to each gene in a group are used to generate a heatmap. The red and blue colours specify up-and down-regulated expressions.

Phytohormones work as central regulators of plant defense (Mine et al., 2018). Intricate mechanisms of phytohormone signaling allow plants to activate appropriate and effective defense responses against pathogens, while maintaining a delicate symbiotic relationship between defense and growth (Ngou et al., 2022). SA induces a defense response against biotrophic pathogens (Kachroo and Kachroo, 2009). Pathogenesis-related proteins, phytoalexins, and hypersensitive responses (HR) are the main defense strategies associated with SA. The DEGs associated with SA biosynthesis were identified and arranged specifically for gene families. These DEGs are comprised of NPR1-like protein 3 (NPR), TGACG-Binding (*TGA*) and pathogenesis-related protein (*PR1*) (**Figures 7A–C** and **Table S3**). One NPR1 protein encoding gene, *Cla97C07G137510*, was found upregulated in response to PM infection in resistant lines. Moreover, two PR1 genes (*Cla97C02G034020*, *Cla97C03G063250*) and one TGA gene (*Cla97C07G048210*) were identified in resistant lines. The genes were upregulated in the R_I vs.. R_CK group. Interestingly, comparison of various groups clearly demonstrated that the majority of SA biosynthesis genes responded in resistant lines after PM infection. Moreover, either these genes were not detected or downregulated in sensitive lines.

Furthermore, ethylene responsive genes were also studied (**Figures 7D, E** and **Table S3**). The analysis revealed that DEGs related to ethylene response factor (*ERF-1/2*) and ethylene-insensitive-3 (*EIN-3*) were identified in comparison groups. Upregulation of the *Cla97C07G136410* (*EIN-3*) gene was observed in the R_I vs.. R_CK group, whereas no differential transcript was observed in other groups. Two *ERF1/2* genes (*Cla97C05G085160* and *Cla97C06G116550*) were identified with upregulated transcripts in both lines after PM infection. In contrast, one *ERF1/2* (*Cla97C05G085160*) gene was downregulated when a mock-treated resistant line was compared with a sensitive line (R_CK vs. S_CK). However, transcripts of other genes, including *CTRI*, *SlMKK*, and *MPK6*, were not detected.

The detailed analysis for JA-relate genes showed that jasmonate-zim-domain protein 3 (*JAZ3*) and MYC2 genes had differential expression in watermelon comparison groups (**Figures 7F, G**). In summary, four *JAZ* genes and one *MYC2* gene were downregulated in R_I vs. R_CK and S_I vs.. R_CK, respectively. Only one *JAZ* gene (*Cla97C07G141340*) was upregulated in R_CK vs. S_CK.

### 3.7 Differential abundance and induced expression of DEGs associated with lignin biosynthesis in response to PM invasion

Pathogen invasion on plants is strongly reacted to by the phenylpropanoid pathway, which encodes the enzymes in the lignin building monolignol units (Yadav et al., 2020). The infection by pathogen to penetrate plant tissues is often escorted by localized appositions, which are majorly identified as lignified materials (Bhuiyan et al., 2008). In our current research, a significant number of genes associated with monolignol biosynthesis processes were identified. The mining of the genes linked with monolignol biosynthesis was performed to differentiate the roles of lignin monomer units in cell wall-mediated defense against PM infection in watermelon. The detailed analysis showed that phenylpropanoid biosynthesis and phenylalanine metabolism are two of the main processes involved in watermelon seedlings’ early-stage resistance to PM invasion. Our results showed that nine sets of genes that are involved in monolignol biosynthesis were identified with differential expression during PM invasion of susceptible and resistant watermelon lines, over the early stages of invasion in leaves. Transcriptome data showed that steady-state levels of *ClPAL, Cl4Cl, ClHCT, ClCCR, ClCAD, ClCCoAOMT, ClCOMT, ClUGT72E, ClCSE*, and *ClCYP73A* transcripts were differentially expressed in leaves during the primary stages of PM invasion. The overall expression in various comparison groups is presented in **Figure 4**. Comprehensive explanations are provided below in the following sub-sections.

#### 3.7.1 Lignin biosynthesis pathway associated differential genes in assembled transcripts

**Figure 8:**
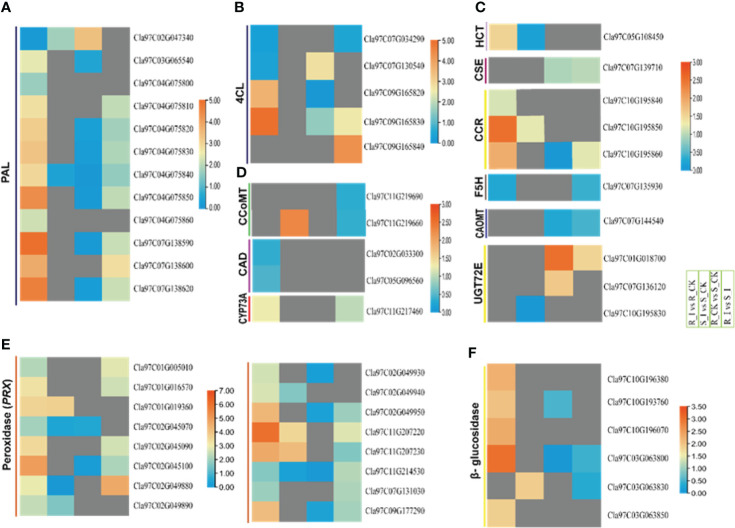
Heatmap of the genes linked to the general phenylpropanoid pathway, peroxidase and beta-glucosidase were identified with differential expression in PM invasion of resistant and susceptible watermelon lines, over early stages of invasion in leaves. Selected DEGs from the *PAL* **(A)**, *4CL* **(B)**, *HCT, CSE, CCR, F5H, CAOMT* **(C)**, *CCoAOMT, CAD, CYP73A* **(D)**, peroxidase (*PRX*) **(E)** and β-glucosidase **(F)** gene families from different comparative combination groups R_I vs. R_CK, S_I vs. S_CK, R_CK *vs.* S_I vs. S_I are presented in form of a heatmap. Log FC values corresponding to each gene in a group are used to generate a heatmap. The red and blue colours specify up-and down-regulated expressions.

In-depth analysis of the filtered DEGs associated with lignin biosynthesis pathways revealed that DEGs from a number of gene families encoding proteins such as *ClPALs, Cl4Cls, ClHCTs*, and *ClCCRs* showed differential trends in various comparison groups. Ten *PAL*s were reported with differential expression levels after PM inoculation in both resistant and non-resistant lines after PM inoculation. In detail, three genes, including *Cla97C04G075850*, *Cla97C07G138590*, and *Cla97C07G138620*, showed sharp upregulation in the resistant line after PM inoculation. Similarly, the R_I vs. S_I comparison showed that four *PAL* genes had higher transcript levels in resistant lines. Other genes, including two *4CL*, one *CYP73A*, three *CCR*, one *HCT*, and genes from the phenylpropanoid pathway, exhibited higher transcript accumulation in resistant lines after PM infection **(** **Figures 8A–D** **)**. Two *4CL* genes (*Cla97C09G165820/30*) were upregulated in the R_I vs. R_CK group, whereas two other genes were upregulated in the R_I vs. S_I group. The analysis showed that no DEGs were detected in the sensitive line in response to PM infection. *CCR* and *HCT* gene analysis also revealed that these genes respond to PM infection in resistant lines. Interestingly, one *CCoMT* and two *CCR* genes showed upregulation in sensitive lines after PM infection. Overall, the analysis reports confirmed that the majority of the genes from lignin biosynthesis pathways responded well to resistance lines in resistance lines and higher transcripts were detected in infected plants of resistance lines when compared to mock-treated plants. A smaller number of genes responded in sensitive lines and only a handful of genes showed upregulation response in sensitive lines **(** **Figures 8A–D** **)**.

#### 3.7.2 Regulation of peroxidase (*PRXs*) and β-glucosidase related DEGs in compatible and incompatible reaction

Among the proteins induced during plant pathogen interactions, plant peroxidases enzymes are well known (Almagro et al., 2008). These proteins belong to a large multigene family that actively participates in the cross-linking of cell wall components and lignin formation. Moreover, these genes actively participate in the biosynthesis of phytoalexins and hypersensitive response (HR) against pathogens. Our current study demonstrated that 28 *PRX*s DEGs in all combination groups were identified (**Table S3**). Except for a few genes specific to groups, the majority of genes were upregulated in the resistance line **(** **Figure 8E** **)**. In group R_I vs. R_CK, 20 *PRX*s genes were upregulated and only eight genes were identified with lower transcript accumulation. Four genes, including *Cla97C02G045100*, *Cla02G049950*, *Cla97C11G207230*, and *Cla97C09G177290*, were reported with a multi-fold increase in transcripts after PM infection. Only three genes were upregulated in the S_I vs. S_CK comparison group. Similarly, the detailed analysis of all β-glucosidase revealed that the majority of genes responded after PM infection in the resistance line. A total 12 number of β-glucosidase genes were identified in four comparison groups (**Table S3**). Most of the genes were upregulated in resistant lines, whereas two genes showed a significantly downregulated pattern in sensitive lines (**Figure 8F**). The transcription levels of five out of twelve genes were significantly higher in the R_I vs. R_CK group, especially the transcript level of *Cla97C03G063800*, which was upregulated multi-fold in infected plants of the resistant line.

### 3.8 Reliability of RNA-seq data by quantitative real-time PCR

**Figure 9:**
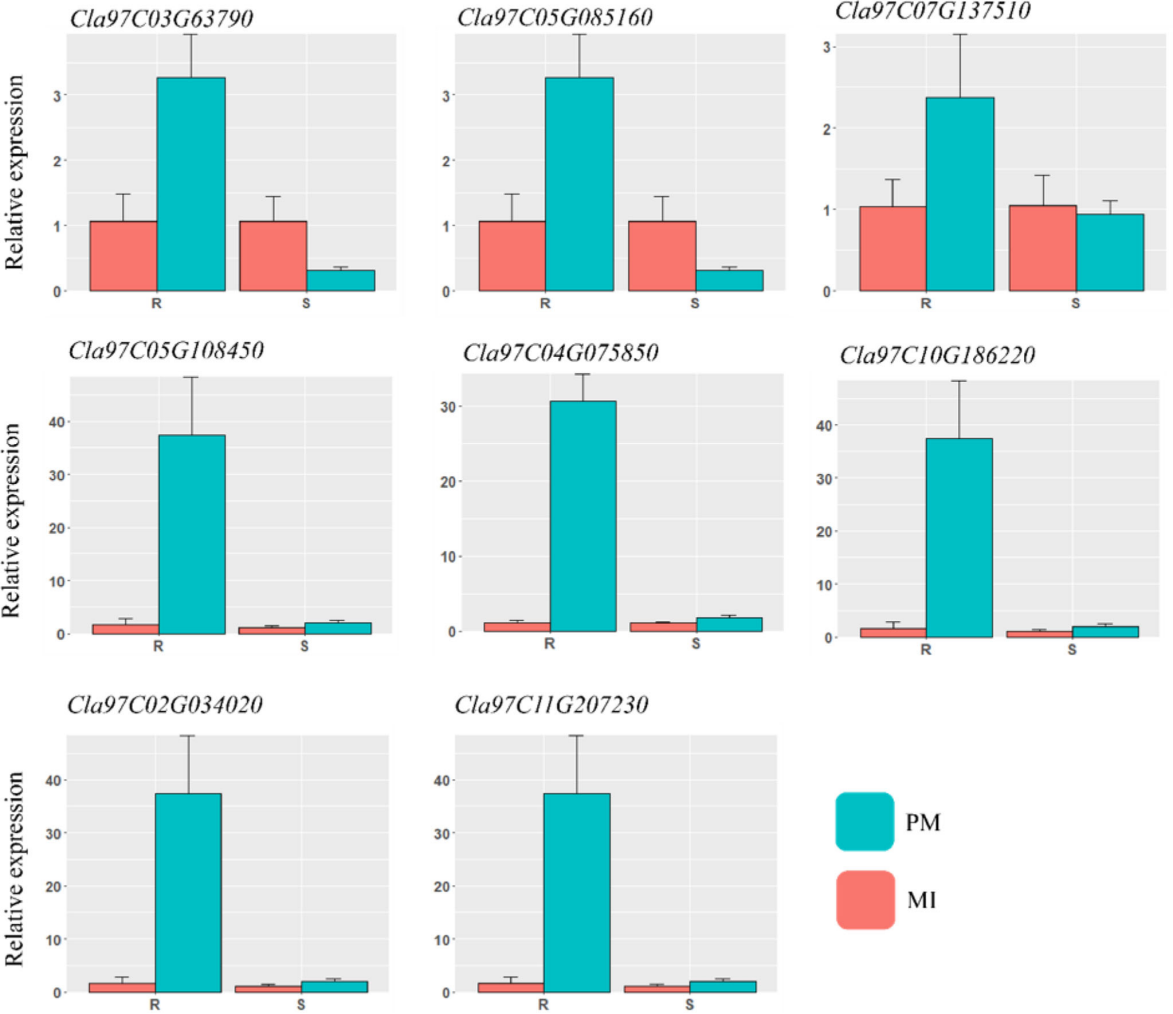
qRT-PCR validation of the various DEGs identified in different defense related pathways. The important candidate genes were selected and analyzed in contrasting watermelon lines mock inoculated (MI) and powdery mildew inoculated (PM).

For reliability of the RNA-seq dataset, a qRT-PCR experiment was carried out with eight DEGs related to various pathways, including lignin biosynthesis, hormones, peroxidase activities, and β-glucosidase. The comparison showed consistency in the transcript accumulation pattern with those found by RNA-sequencing analysis. The qRT-PCR results are presented in **Figure 9**. Gene specific primers were designed by using Primer3plus web tool (https://www.primer3plus.com/) software and the respective primers are listed in **Table S4**. The positive trend in both data in all mock inoculated and PM samples showed the reliability of RNA-sequencing.

## 4 Discussion

Powdery mildew is a widespread disease of many cucurbits caused by a biotrophic fungus (Kousik et al., 2018). The PM fungus invades watermelon during multiple stages of growth and development, causing direct effects on fruit quality and yield (Kousik et al., 2019; Yadav et al., 2021a). The current study provides insight on early-stage incompatible and compatible interactions in watermelon against PM race ‘2F’ fungus through the use of physiological, microscopic, and transcriptomic analysis.

To determine the precise expression changes induced by *P. xanthii* race 2F in both lines during the early stage of infection, differential expression analysis was performed on powdery mildew race 2F treatments (I) vs. mock samples (CK) of both contrasting genotypes, i.e., ZXG1755 (R) and ZXG1996 (S) over both time points. In response to *P. xanthii* race 2F infection, both S and R showed significant transcriptional regulation. Moreover, our study portrayed strong evidence indicating the important role of a complex network associated with lignin biosynthesis and phytohormone related downstream mechanisms that are responsible for incompatible interaction between PM and watermelon resistance lines. In our present study, 1718 and 1115 genes in R_I vs. R_CK and S_I vs. S_CK were identified. These DEGs were predominantly abundant in the KEGG pathway of the plant hormone and transduction, phenylpropanoid biosynthesis and phenylalanine metabolism, whereas GO terms of defense response, response to pathogens and chitin responsive were predominant in resistant lines, evidencing significant defense mechanisms and differences in the basal gene expression levels between these contrasting lines. Similar findings were reported in other crops, which also evidenced similar findings.

### 4.1 Phytohormone and signal transduction pathway in relevance to resistance response against PM invasion

Auxins, cytokinins, gibberellins, jasmonic acid, salicylic, acid ethylene, abscisic acid, and brassinosteroid are phytohormones that respond to stress by acting synergistically and antagonistically in a process known as signaling cross talk (Bostock, 2005; Sood et al., 2021; Gilroy and Breen, 2022). These phytohormones respond to environmental stimuli and developmental cues by harmoniously coordinating with one another (Dopp et al., 2021; Fidler et al., 2022). All plant defensive responses are the consequence of the interaction of numerous genes and gene families that coordinate in a network (Pandey and Somssich, 2009; Kumar et al., 2021). Through the regulation of genes, several phytohormones are recognized to have a significant part in multiple processes (Figueroa-Macias et al., 2021; Zhao et al., 2021; Benjamin et al., 2022). Furthermore, plants maintain homeostasis and adjust to environmental changes. This is only feasible because different phytohormones interact effectively and systematically, enabling plants to maintain a vital balance between growth and environmental reaction (Monson et al., 2022). In the present research, five ABA, four SA, and three ET signaling genes were identified, which were upregulated in the incompatible reaction of ZXG1755 against PM infection. In the ethylene signaling pathway, *EIN3* works as a key regulator in ethylene signaling. *Cla97C07G136410* (*EIN3*) was upregulated in the resistant line after PM inoculation. Two ET response factor 1-homologous genes (*ERF1/2, Cla97C05G085160*, and *Cla97C06G116550*) were up-regulated in ZXG1755 at 48 hpi. These *ERF* genes are found to interact with the GCC box and facilitate pathogenic resistance in plants (Mizuno et al., 2006; Schwessinger et al., 2015; Kunwar et al., 2018). Some studies evidence that overexpression of *ERF1* and *ERF2* coordinates and increases the transcription level of defense genes including *PDF1.2* and *b-CHI* against necrotrophic fungus (Berrocal-Lobo and Molina, 2004; McGrath et al., 2005).

Plant defense against pathogens is facilitated by PR genes, which are well-known for this significant role in plant defense (Bozbuga, 2020; Kapoor et al., 2021; Sood et al., 2021). Transgenic tobacco plants with the PR-1 gene overexpressed showed improved resistance to *P. parasitica* compared to control plants, while transgenic tobacco plants with the *PR-1* gene silenced had increased susceptibility to *P. parasitica*. Inferring, PR-1 might operate as a positive regulator of plant resistance to *P. parasitica*. Systemic acquired resistance (SAR) is connected to *PR-1* increased expression, which is likewise considered as a SA-based defense mechanism (Peng and Huang, 2005; Knoth and Eulgem, 2008). Interestingly, in our current work, we reported that many genes from the biosynthesis pathway of SA were upregulated during the PM response in resistant lines. *Cla97C09G177290*, which is annotated as a *PR1* gene, increased multi-fold in resistant lines after PM inoculation.

### 4.2 Lignin biosynthesis pathways related genes shows distinct responses to PM

**Figure 10:**
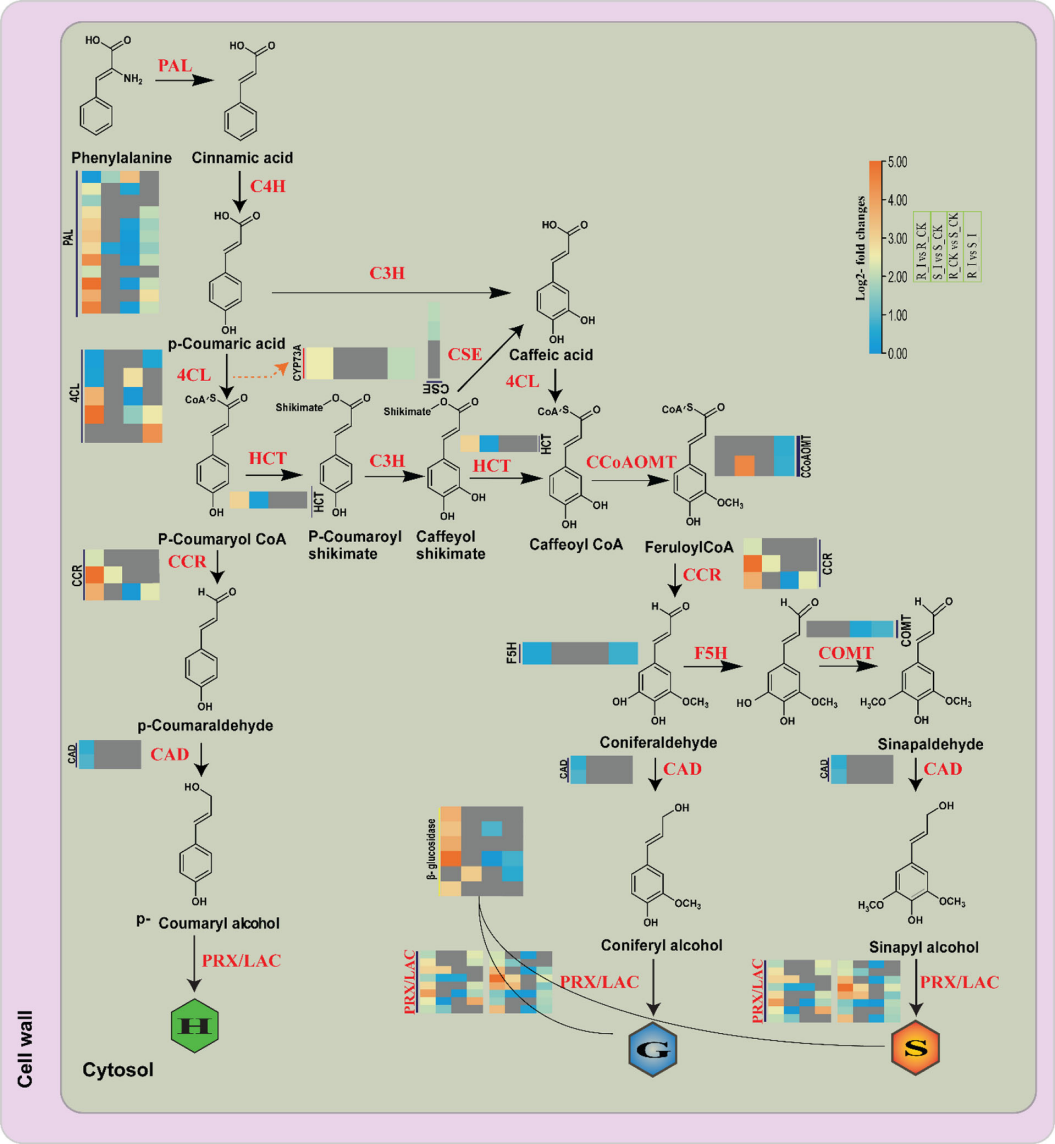
Differential expression of genes related with the lignin biosynthesis pathway in different comparison groups of resistance and susceptible watermelon lines during an early stage of PM infection. The Heat map was generated using log2FC, where the red and blue colors specify up-and down-regulated expressions in comparison groups such as R_CK vs. R_I, S_CK vs. S_I, S_CK vs. R_CK and S_I vs. R_I (Left to right/bottom to top). PAL, phenylalanine ammonia-lyase; F5H, coniferaldehyde/ferulate5 hydroxylase; CCR, cinnamoyl-CoA reductase; COMT, caffeic acid/5-hydroxyferulic acid O-methyltransferase; CSE, caffeoyl shikimate esterase; CCoAOMT, caffeoyl-CoAO methyltransferase; C4H, cinnamic acid4 hydroxylase; CAD, (hydroxy)cinnamyl alcohol dehydrogenase; C3′H, p-coumaroyl shikimate3′ hydroxylase; 4CL, 4-hydroxycinnamoyl-CoA ligase; HCT, hydroxycinnamoyl-CoA: shikimate/quinate hydroxycinnamoyl transferase.

General phenylpropanoid pathways and lignin-specific pathways are identified as defense regulators against pathogen invasion in many plant species (Xie et al., 2018; Ha et al., 2021). Available literature suggests that the phenylpropanoid biosynthesis genes strongly respond to pathogen attack in many plants (Sattler and Funnell-Harris, 2013; Gallego-Giraldo et al., 2018; Cardoni et al., 2022) Resistance to Bgt in Tibetan barley, resistance to leaf blight in rice, tomato early blight, and white rust disease in chrysanthemum all had a strong relationship with phenylpropanoid pathway defense mechanisms. Phenylpropanoids, including a number of compounds including phenolic compounds, flavonoids and lignin, are the outcome of different downstream pathways (Dixon et al., 2002). Detailed analysis of DEG associated with the phenylpropanoid pathway and monolignol biosynthesis showed that a number of genes had induced expression in both incompatible and compatible lines in the early stages of PM infection (**Figure 10**). Our analysis is in agreement with reports from other sources on the induction of genes coding the protein which are crucial for monolignol biosynthesis in plants after pathogen infections in a variety of plant species. A previous report in wheat showed that all the transcripts of monolignol genes were accumulated at an early stage of powdery mildew infection in resistant lines (Bhuiyan et al., 2007; Bhuiyan et al., 2008; Bhuiyan et al., 2009). Similarly, the findings of barley–powdery mildew early-stage interactions revealed upregulation of defense-related genes (Caldo et al., 2004). In our study, PAL genes from R_I *vs.* R_CK showed upregulated genes. The upregulation trend of *PAL* genes is in line with the previous findings reported by (Zierold et al., 2005), where a rise in mRNA transcript with PAL enzyme was reported in barley upon PM infection. By facilitating the production of CoA ester, *4CL* plays a variety of significant roles during environmental stresses experienced by plants (Xiang et al., 2020; Zhang et al., 2022). In our study, some *4CL* genes showed induced expression in the R_I *vs.* R_CK and R_I vs. S_I groups (**Figure 10**), suggesting that our isoforms of 4CL may have a positive role in the resistance mechanism. Some O-methyltransferase genes can potentially act in various branches of the phenylpropanoid pathway. *COMT* and *CCoAOMT* belong to the O-methyltransferase (OMT) family, which further methylates a number of secondary metabolites that includes flavonoids, phenylpropanoids, and alkaloids, which have defense potential against pathogens (Chang et al., 2021). For instance, the *TaCOMT-3D* gene of wheat confirms resistance against eyespot disease. In contrast, our findings suggested that genes in the O-methyltransferase (OMT) family had no response in the resistant line, whereas one *CCoMT* (*Cla97C11G9660*) was upregulated in the sensitive line after inoculation. *CYP73A* is an important gene that encodes trans-Cinnamate 4-hydroxylase (*C4H*) and is involved in the catalyzation steps in the phenylpropanoid pathway (Batard et al., 1997; Zhang et al., 2020). It was reported that induction of the CYP73A gene after wounding and chemical treatment in Helianthus tuberosus leads to an increase in the accumulation of C4H genes (Batard et al., 1997). Similarly, in our study, one CYP73A (*Cla97C11G217460*) was induced in resistance lines in stress conditions. Under PM stress conditions, some more genes, such as one hydroxycinnamoyltransferase (*HCT*) and three cinnamoyl CoA Reductase (*CCR*) genes, were found to be related to the resistance behavior of ZXG1755. Prior research demonstrated that *CCR* genes contribute to plant defense by enhancing the accumulation of monolignols, the fundamental building block of the cell wall (Tronchet et al., 2010). For instance, the plant pathogen *Xanthomonas campestris* pv. campestris caused the induction of Arabidopsis *AtCCR2*, which may have an important role in plant pathogen defense. Similarly, one *CCR* (*OsCCR1*) gene in rice was identified to have induced expression of sphingolipid elicitor, evidencing the defense role of the gene against *Xanthomonas oryza* (Kawasaki et al., 2006) Furthermore, a study has shown that *CCR* genes also contribute to UV stress resistance.

### 4.3 Peroxidase and in early-stage defense reaction against PM

Peroxidases (EC1.11.1.7) are among the important inducible proteins that induces post inoculation in defense response (Almagro et al., 2008). These genes belong to a large multigene family having important role in various activities including lignin formation, suberin synthesis, cross-linking of cell wall, RNS and ROS activities and HR response in resistant plants (Zhao et al., 2005; Garcia-Brugger et al., 2006). *PRX* genes actively participate in oxidation of monolignol units to form lignin polymer (Gao et al., 2021). Previous studies clearly evidenced that POD activities were increase in plants post infection. For instance, the expression of *PRX* genes were induced by Bgt attack in mesophyll and epidermis wheat tissues (Liu et al., 2005). Similarly, higher peroxidase activities were reported in citrus plants infected with *Pichia galeiformis* (Chen et al., 2021). A recent report in melon showed that number of genes encoding peroxidase portions were induced in resistant lines and their response in sustainable lines was neutral after PM infection (Zhu et al., 2018). In this study, total 28 genes DEGs were identified in resistant and sensitive line, and 15 and 7 *PRX* genes were significantly upregulated in sensitive and resistant lines, respectively. Similar to our studies a higher POD activity in cotton was observed against infection by *Verticillium dahlia* (Xu et al., 2011). Significant induction of *PRX* genes specifically in resist lines make them potential candidate that have contribution in defense mechanism against PM infection.

## 5 Conclusion

In the present study, a comparative transcriptome profiling of gene expression alterations was undertaken between resistant line ZXG1755 and susceptible line ZXG1996. Our analysis identified some major pathways and certain number of genes, which were highly responsive in resistant lines in comparison to sensitive lines after PM inoculation. Based on out study, resistant lines might have lignin and hormone signaling based downstream disease defense mechanisms. The comparative studies identified number of candidate genes associated with PR proteins, phenylpropanoids, defense phytohormones and lignin biosynthesis related defense *via* atypical mechanisms. qRT-PCR was also used to validate several candidate genes with potential pathogen defense roles in response to watermelon-PM infection. Future functional investigation of these PM-responsive genes is anticipated to contribute to a greater comprehension of the molecular mechanisms underlying wheat’s pathogen defense. This study provides a theoretical foundation for the production of watermelon cultivars resistant to powdery mildew, in addition to allowing the identification and analysis of other candidate genes and the investigation of the detailed mechanisms underlying watermelon defense responses against PM.

## Data availability statement

The datasets presented in this study can be found in online repositories: https://www.ncbi.nlm.nih.gov/bioproject/881394, PRJNA881394.

## Author contributions

VY and XZ conceived and directed the original research plan and wrote the manuscript. VY, ZW, and YG executed data analysis and carried out experiments. All authors contributed to the article and approved the submitted version.

## Funding

Modern Agroindustry Technology Research System of China (CARS-25), Agricultural Science and Technology innovation Drive project of Shaanxi (NYKJ-2021-YL(XN)42), Key R&D project of Yangling Seed Industry Innovation Center (Ylzy-sc-01), Innovative demonstration projects for high-quality agricultural development and ecological protection (NGSB-2021-7).

## Acknowledgments

The authors would like to thank Zhengzhou Fruit Research Institute, Chinese Academy of Agricultural Sciences, Zhengzhou for providing us the plant seed used in the current study.

## Conflict of interest

The authors declare that the research was conducted in the absence of any commercial or financial relationships that could be construed as a potential conflict of interest.

## Publisher’s note

All claims expressed in this article are solely those of the authors and do not necessarily represent those of their affiliated organizations, or those of the publisher, the editors and the reviewers. Any product that may be evaluated in this article, or claim that may be made by its manufacturer, is not guaranteed or endorsed by the publisher.
